# Effects of forest bathing (shinrin-yoku) on serotonin in serum, depressive symptoms and subjective sleep quality in middle-aged males

**DOI:** 10.1265/ehpm.22-00136

**Published:** 2022-11-02

**Authors:** Qing Li, Hiroko Ochiai, Toshiya Ochiai, Norimasa Takayama, Shigeyoshi Kumeda, Takashi Miura, Yoichiro Aoyagi, Michiko Imai

**Affiliations:** 1 Department of Rehabilitation Medicine, Graduate School of Medicine, Nippon Medical School; 2 Department of Plastic and Reconstructive Surgery, Laboratory of Regenerative Medicine, Division of Hearing and Balance Disorder, National Institute of Sensory Organs, National Hospital Organization Tokyo Medical Center; 3 Forest Baubiologie Studio Inc.; 4 Forestry and Forest Products Research Institute, Forest Research and Management Organization; 5 Nagano Prefectural Kiso Hospital; 6 Agematsu Town Office Industry & Tourism Department; 7 INFOM (International Society of Nature and Forest Medicine)

**Keywords:** Depression, Forest bathing, Middle-aged males, Oguri-Shirakawa-Azumi sleep inventory MA version (OSA-MA), POMS, Serotonin, Shinrin-yoku, Sleep

## Abstract

**Background:**

We previously found that a forest bathing (shinrin-yoku) program significantly reduced the scores for depression, anxiety, anger, fatigue, and confusion and increased the score for vigor in the profile of mood states (POMS) test and showed a potential preventive effect on the depressive status in both males and females. In the present study, we investigated the effects of a forest bathing program on the level of serotonin in serum, depressive symptoms and subjective sleep quality in middle-aged males.

**Methods:**

Twenty healthy male subjects aged 57.3 ± 8.4 years were selected after obtaining informed consent. These subjects took day trips to a forest park, the birthplace of forest bathing in Japan named Akasawa Shizen Kyuyourin, Agematsu, Nagano Prefecture (situated in central Japan), and to an urban area of Nagano Prefecture as a control in June 2019. On both trips, they walked 2.5 km for 2 hours each in the morning and afternoon on Saturday and Sunday, respectively. Blood was sampled in the afternoon before and after each trip. Concentrations of serotonin and lactic acid in serum were measured. The POMS test and a questionnaire for subjective sleep quality were conducted before and after the trips. Ambient temperature and humidity were monitoring during the trips. The Ethics Committees of the Nippon Medical School and Nagano Prefectural Kiso Hospital approved this study.

**Results:**

The forest bathing program significantly increased level of serotonin in serum, and significantly increased the score for vigor and decreased the score for fatigue in the POMS test. The forest bathing program also improved the sleepiness on rising and feeling refreshed (recovery from fatigue) in the Oguri-Shirakawa-Azumi sleep inventory MA version (OSA-MA).

**Conclusions:**

Taken together, the present study suggests that forest bathing may have potential preventive effects on depression (depressive status).

## Background

The forest environment has long been enjoyed for its quiet atmosphere, beautiful scenery, calm climate, pleasant aromas, and clean fresh air. Stress is a keyword to understand the reason why forest bathing is attracting attention in Japan. In 1980s, the word ‘technostress’ was coined to describe unhealthy behaviour around new technology. Technostress can arise from all manner of everyday usage, like checking cellular phone constantly, compulsively sharing updates and feeling that you need to be continually connected. Symptoms run from anxiety, headaches, depression, mental fatigue, eye and neck strain to insomnia, frustration, irritability and loss of temper [1, 2]. According to the Ministry of Health, Labour and Welfare of Japan, the percentage of workers with anxiety and stress was more than 50% in 1982 [3]. Based on the above background, in Japan, a national health programme for forest bathing or shinrin-yoku began to be introduced in 1982 by the Forest Agency of Japan for the stress management of workers in Japan [3]. Shinrin-yoku is translated into forest bathing in English. *Shinrin* in Japanese means ‘forest’, and *yoku* means ‘bath’. Therefore, *shinrin-yoku* means bathing in the forest atmosphere, or taking in the forest through our senses. This is not exercise, or hiking, or jogging. It is simply being in nature, connecting with it through our sense of sight, hearing, taste, smell and touch [1–4]. Since forests occupy 67% of the land in Japan, forest bathing is easily accessible in Japan [3]. Forest bathing as a recognized relaxation and/or stress management activity and a method of preventing diseases and promoting health is becoming a focus of public attention in Japan [3]. According to a public opinion poll conducted in Japan in 2003, 25.6% of respondents had participated in a forest bathing trip, indicating its popularity in Japan [5]. Currently, the terms of “Shinrin-yoku” and “Forest bathing” are internationally accepted because both “Shinrin-yoku” and “Forest bathing” are the titles of English books [1, 2] and books in other languages [6, 7].

We previously found that forest bathing reduces stress hormones such as adrenaline and noradrenaline in urine in males and/or females in 2-night/3-day forest bathing trips [8, 9], and reduces stress hormone, cortisol in serum in males in a day trip [10]. Forest bathing also reduces sympathetic nervous activity and increase parasympathetic nervous activity and showed the relaxing effect both in male and female subjects [11–16]. In addition, in the profile of mood states (POMS) test, forest bathing reduces the negative emotions such as tension–anxiety, anger, depression, fatigue and confusion and increase in feelings of vigor and showed the relaxing effect both in male and female subjects [3, 4, 9–16]. These findings suggest that forest bathing may have a potential preventive effect on depressive status. On the other hand, it has been reported that patients with major depressive disorder (MDD) show lower level of serotonin in serum [17–21]. Moroianu et al. [22] also reported that there are statistically significant inverse correlations between the levels of serotonin in serum and the values calculated for degree of depression depending on the Beck Depression Inventory and degree of anxiety depending on the Hamilton A questionnaire scale, respectively, in patients with Type 2 diabetes who show anxiety and depression. However, there is no study on the effects of forest bathing on serotonin in serum in humans.

Based on the above background, we hypothesize that forest bathing may increase the level of blood serotonin, thus, in the present study, we investigated the effects of forest bathing on the serotonin level in serum in middle-aged males without major depressive disorder.

## Subjects and methods

### Subjects

In our published studies previously, the numbers of subjects were 9–20 [3, 4, 8–16]. Based on the results of previous studies, in the present study, twenty healthy male subjects without major depressive disorder, who ranged in age from 41–69 years (mean ± standard deviation (SD): 57.3 ± 8.4 years), were selected for the present study as shown in Table 1. Written informed consent was obtained from all subjects after a full explanation of the study procedures. None of the subjects had any symptoms of disease, used drugs that might have affected the results, or were taking any medication at the time of the study. The subjects consumed the same number of calories during the two trips. To control for the effects of alcohol, the subjects did not consume alcohol during the study period. This study was conducted under the Declaration of Helsinki. The Ethics Committees of the Nippon Medical School and Nagano Prefectural Kiso Hospital approved this study.

**Table 1:** Information of the subjects

**No**	**Sex**	**Age**	**Height ** **(cm)**	**Body weight ** **(kg)**	**BMI**	**Sleep time (h) ** **Before**	**Sleep time (h) ** **Day 1**	**Sleep time (h) ** **Day 2**
1	Male	41	165	85	31.2	6	6.5	7
2	Male	47	178	95	30.0	6	8	8
3	Male	50	172	80	27.0	5	5	6
4	Male	52	173	104	34.7	7	7	8
5	Male	55	164	74	27.5	6	5	5
6	Male	67	170	67	23.2	6.5	8	8
7	Male	61	177	76	24.3	8	8	7
8	Male	61	171	60	20.5	4.5	5	5
9	Male	66	160	64	25.0	6	5.5	7.3
10	Male	69	155	58	24.1	7	6	7
11	Male	47	164	68	25.3	7	6	7
12	Male	49	173	63	21.0	6	6.5	5.8
13	Male	51	171	65	22.2	8	8	8.3
14	Male	53	188	94	26.6	6	7	6.8
15	Male	57	173	67	22.4	6	9	7.5
16	Male	59	180	88	27.2	5.5	7	7
17	Male	61	169	67	23.5	7	8	7.5
18	Male	63	168	63	22.3	6.5	6	7
19	Male	68	178	58	18.3	7	6.7	6.5
20	Male	69	183	71	21.2	6.5	6	7
Mean		57.3	171.6	73.4	24.9	6.4	6.7	6.9
SD		8.4	7.9	13.4	4.0	0.9	1.2	0.9
n		20	20	20	20	20	20	20

Sleep time (h) Before: Sleep time before the experiment

Sleep time (h) Day 1: Sleep time during the experiment on day 1

Sleep time (h) Day 2: Sleep time during the experiment on day 2

### Walking in a forest environment and an urban area

The subjects took 3-day trip to a forest park named Akasawa Shizen Kyuyourin (Akasawa Natural Recreation Forest), Agematsu, Nagano Prefecture (situated in central Japan), which is the birthplace of forest bathing in Japan and to an urban area of Nagano Prefecture as a control where there were almost no trees in June 2019.

On the first day (Friday), all participants departed from Tokyo in the morning and arrived at a hospital near Akasawa Shizen Kyuyourin and blood samples were taken at 4 pm for the measurements before the forest bathing. Then they stayed at a hotel near the hospital.

On the second day (Saturday), ten of the 20 subjects were selected at random and assigned to the urban site and the other ten subjects were assigned to the forest site. In both trips, they walked 2.5 km for 120 min in the morning (10 am–12 pm) and afternoon (1 pm–3 pm), respectively for a total 5.0 km per day on Saturday. During the walking, the subjects took a short break for two times. The blood samples were taken at 4 pm at the same hospital after the walking in forest and unban sites. Then they stayed at the same hotel.

On the third day (Sunday), subjects switched field sites. The experiment protocol was the same with the second day (Saturday). The blood samples were taken at 4 pm at the same hospital after the walking in forest and unban sites. Then the experiment was finished and the subjects returned to Tokyo.

During these 3 days, all participants stayed in identical single rooms at the same local hotel. Intake of all foods and physical activity were controlled, and smoking and drinking alcoholic or caffeinated beverages were prohibited. All participants took the same diet and there was no any variation in the type of food they consumed during the experiments. Daily physical activity of the subjects was monitored with a pedometer [4, 8–10, 12, 13].

Ambient temperature and humidity were monitoring during the trips as reported previously [13]. On Saturday, it was cloudy and sometimes rainy in urban areas, and rainy and sometimes cloudy in forest areas. It was raining in both urban and forest areas on Sunday. On both days, the urban environmental temperature was higher than in the forest areas, whereas the humidity in the forest areas was higher than in the urban areas.

### Physiological and psychological indices

The concentration of serotonin in serum was measured by the Bio Medical Laboratories (BML), Inc. in Tokyo, Japan with high performance liquid chromatography (HPLC) method. The range of measurement is 81.0–262.0 ng/mL. To monitor the physical activity of subjects, lactic acid concentration in serum was also measured in the laboratory of Nagano Prefectural Kiso Hospital by enzyme method with a range of 5.0–20.0 mg/dL. Both the detection rates of serotonin and lactic acid concentrations are 100% in the present study. The POMS test was conducted before and after the trips [3, 4, 9–13]. Subjective sleep quality was assessed using the Oguri-Shirakawa-Azumi sleep inventory MA version (OSA-MA) before and after the forest bathing and city walking in the morning. This sleep questionnaire has been standardized to assess the sleep quality of middle-aged and elderly Japanese people [23]. The OSA-MA consists of 16 items measured according to a four-point rating scale and consolidated into the following five factors: sleepiness on rising, initiation and maintenance of sleep, frequent dreaming, feeling refreshed (recovery from fatigue), and sleep length. The OSA-MA scores were calculated as corrected (Zc) scores, with higher scores indicating better quality of sleep [23].

### Statistical analysis

If the samples are of equal variance, a paired t-test can be used. In this study, we performed a variance test (F-test) before performing a t-test. We confirmed that the data were of equal variance. Thus, paired t-test was used to compare the differences between urban and forest environments in walking steps, physical activity, lactic acid concentrations, the level of serotonin in serum, and the scores in the POMS test. Paired t-test was also used to compare the differences in subjective sleep quality (sleepiness on rising, feeling refreshed) between before and after trips. The analyses were performed with the Microsoft Excel software package for Windows. The significance level for p values was set at <0.05.

## Results

### Walking steps and physical activity during the forest bathing and urban area walking

Walking steps are 11949 ± 2389 (mean ± SD) steps in forest bathing and 11072 ± 1896 steps in urban area walking, respectively. Physical activities are 382.0 ± 113.6 kcal in forest bathing and 369.5 ± 113.5 kcal in urban area walking, respectively.

**Fig. 1:**
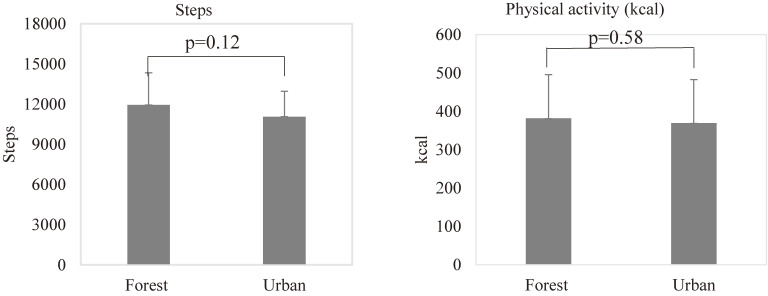
Walking steps and physical activity during the forest bathing and urban area walking. “Forest” means after walking in the forest (forest bathing) and “Urban” means after walking in the urban area. Data are presented with mean + SD, p = 0.12, forest vs urban for walking steps; p = 0.58, forest vs urban for physical activity by paired t-test (n = 20).

As shown in Fig. 1, there was no significant difference in walking steps (p = 0.12) and physical activity (p = 0.58) between the forest bathing and urban area walking.

### Lactic acid concentrations in serum during the forest bathing and urban area walking

Lactic acid concentrations in serum are 6.03 ± 1.84 mg/dL (mean ± SD) in forest bathing and 6.63 ± 1.79 mg/dL in urban area walking, respectively. The information of detailed basic statistics of lactic acid are shown in Table 2.

**Table 2:** Information of basic statistics of lactic acid (mg/dL)

	**After city walking**	**After forest bathing**
Mean	6.63	6.03
Minimum	3.70	2.50
Quartile 25%	5.20	5.20
Median	6.30	6.05
Quartile 75%	8.30	6.73
Maximum	9.60	10.00

**Fig. 2:**
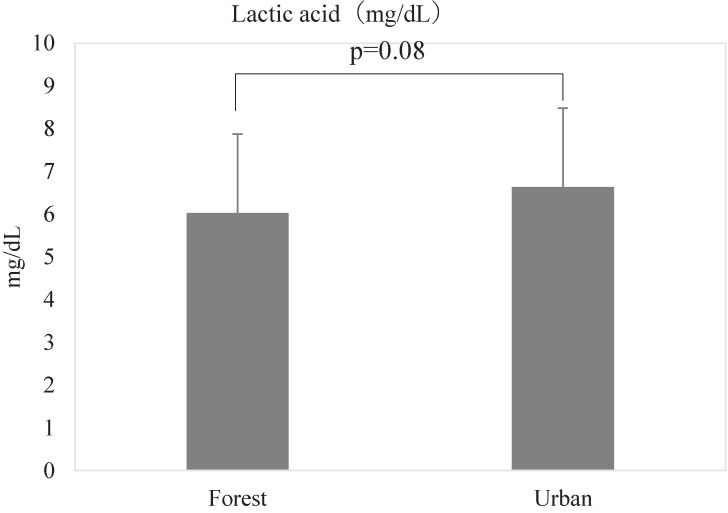
Lactic acid concentrations in serum during the forest bathing and urban area walking. “Forest” means after walking in the forest (forest bathing) and “Urban” means after walking in the urban area. Data are presented with mean + SD, p = 0.08, forest vs urban by paired t-test (n = 20).

As shown in Fig. 2, there was no significant difference in lactic acid concentrations between the forest bathing and urban area walking (p = 0.08).

### Effect of forest bathing on serotonin in serum

The concentrations of serotonin in serum are 78.67 ± 23.65 (mean ± SD) ng/ml before the forest bathing and urban walking, 82.13 ± 28.66 ng/ml after the urban area walking, and 87.46 ± 28.01 ng/ml after the forest bathing, respectively. The information of detailed basic statistics of serotonin are shown in Table 3.

**Table 3:** Information of basic statistics of serotonin (ng/ml)

	**Before**	**After city walking**	**After forest bathing**
Mean	78.67	82.13	87.46
Minimum	43.50	46.80	47.00
Quartile 25%	63.60	63.28	61.83
Median	77.05	76.70	84.30
Quartile 75%	89.23	93.95	103.45
Maximum	133.60	159.20	149.80

**Fig. 3:**
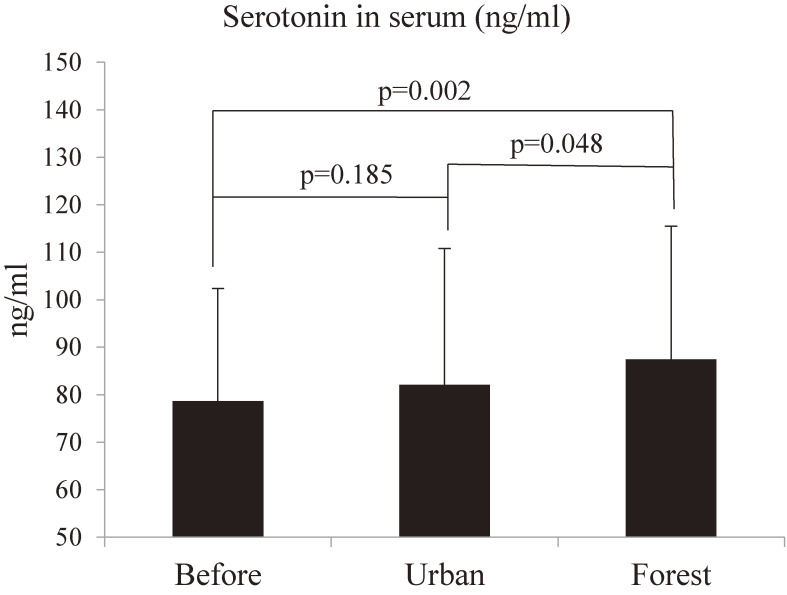
Forest bathing significantly increased the level of serotonin in serum in male subjects. Data are presented with mean + SD, p = 0.002, forest vs before, p = 0.048, forest vs urban, p = 0.185 urban vs before by paired t-test (n = 20) “Before” means before the walking in the urban area or before the forest bating. “Urban” means after walking in the urban area. “Forest” means after walking in the forest (forest bathing).

As shown in Fig. 3, both urban walking and forest bathing increased the level of serotonin in serum; however, there is a significant increase after forest bathing (p = 0.002), but not after the urban area walking (p = 0.185). In addition, there is a significant difference between after forest bathing and after urban area walking (p = 0.048), indicating that forest bathing significantly increased the level of serotonin in serum compared to an urban area walking.

### Effect of forest bathing on depressive symptoms in POMS test

The scores of vigor in the POMS test are 52.45 ± 9.80 (mean ± SD) after forest bathing and 45.85 ± 12.60 after urban area walking, respectively. The score of fatigue in the POMS test are 42.55 ± 6.60 after forest bathing and 47.15 ± 9.72 after urban area walking, respectively.

**Fig. 4:**
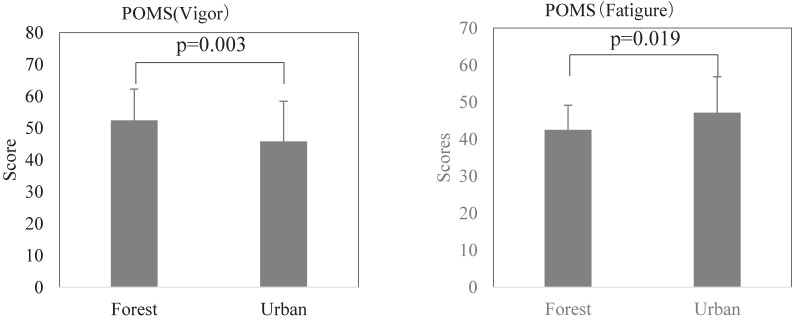
Effect of forest bathing on vigor and fatigue in the POMS test. “Forest” means after walking in the forest (forest bathing) and “Urban” means after walking in the urban area. p = 0.003 forest vs urban for vigor, p = 0.019 forest vs urban for fatigure by paired t-test (Mean + SD, n = 20)

As shown in Fig. 4, the forest bathing significantly increased the score for vigor (p = 0.003) and decreased the score for fatigue (p = 0.019) in the POMS test compared with urban area walking.

### Effect of forest bathing on subjective sleep quality

**Fig. 5:**
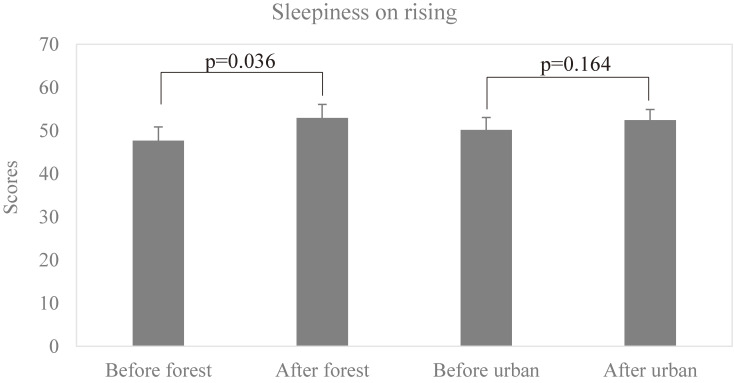
Effect of forest bathing on subjective sleep quality (sleepiness on rising). p = 0.036, between before and after forest bathing, p = 0.164 between before and after walking in the urban area by paired t-test (Mean + SD, n = 10). “Before forest” means before the forest bathing, “After forest” means after the forest bathing, “Before urban” means before the walking in the urban area, and “After urban” means after the the walking in the urban area.

The scores of sleepiness on rising are 47.70 ± 9.52 (mean ± SD) before forest bathing and 52.97 ± 9.37 after forest bathing, respectively, and 50.13 ± 8.75 before urban area walking and 52.43 ± 7.33 after urban area walking, respectively. As shown in Fig. 5, forest bathing significantly improved on sleepiness on rising (p = 0.036) assessed by the OSA-MA.

**Fig. 6:**
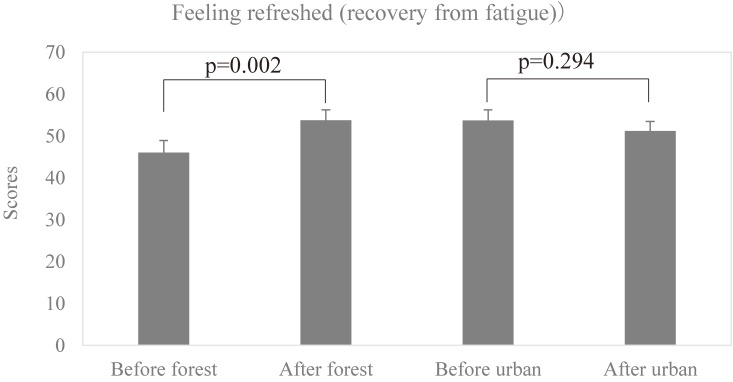
Effect of forest bathing on subjective sleep quality (feeling refreshed, recovery from fatigue). p = 0.002, between before and after forest bathing, p = 0.294 between before and after walking in the urban area by paired t-test (Mean + SD, n = 10). “Before forest” means before the forest bathing, “After forest” means after the forest bathing, “Before urban” means before the walking in the urban area, and “After urban” means after the walking in the urban area.

The scores of feeling refreshed (recovery from fatigue) are 46.05 ± 8.73 before forest bathing and 53.77 ± 7.41 after forest bathing, respectively, and 53.71 ± 7.72 before urban area walking and 51.18 ± 6.90 after urban area walking, respectively. As shown in Fig. 6, forest bathing significantly improved feeling refreshed (recovery from fatigue) (p = 0.002) assessed by the OSA-MA. However, urban area walking did not improve the subjective sleep quality.

On the other hand, both forest bathing and urban area walking did not affect initiation and maintenance of sleep, frequent dreaming and sleep length assessed by the OSA-MA in male subjects.

## Discussion

We previously found that forest bathing reduces stress hormones such as adrenaline and noradrenaline in urine, reduces sympathetic nervous activity and the negative emotions such as tension–anxiety, anger, depression, fatigue and confusion in the POMS test and increase in feelings of vigor and parasympathetic nervous activity and showed the relaxing effect both in male and female subjects [3, 4, 8–16]. These findings suggest that forest bathing may have potential preventive effect on depressive status. On the other hand, the aetiology of major depressive disorder (MDD) in recent decades has been referred to the pathophysiology of the serotonin system [24]. It has been reported that patients with MDD show lower level of serotonin in serum [17–22]. Serotonin concentration was significantly lower in the patients with severe atopic dermatitis, and there was an adverse relation between the serotonin concentration and the score of depression, the features not noticed in the control group [20, 22]. However, there is no study on the effects of forest bathing on serotonin in serum in humans so far.

In the present study, we found that walking in a forest park (forest bathing/shinrin-yoku) significantly increased the concentration of serotonin in serum in the middle-aged males without MDD compared with walking in an urban area. This is a new finding on the effect of forest bathing on human health. Moroianu et al. [22] reported that the levels of serotonin in serum in patients with Type 2 diabetes who show anxiety and depression are 70.77 ± 46.23 µg/L (mean ± SD, n = 48) measured by HPLC. The concentrations are slightly lower than our subjects which are 78.67 ± 23.65 (mean ± SD, n = 20) ng/ml, but at comparable levels. It is very known that patients with MDD show sleep disorders and sleep disturbance is a common and key symptom that affects most of patients with MDD [25, 26]. Thus, we also investigated the effect of forest bathing on subjective sleep quality by the questionnaire of OSA-MA before and after the forest bathing and city walking in the morning. This sleep questionnaire has been standardized to assess the sleep quality of middle-aged and elderly Japanese people [23]. The OSA-MA consists of 16 items measured according to a four-point rating scale and consolidated into the following five factors: sleepiness on rising, initiation and maintenance of sleep, frequent dreaming, feeling refreshed (recovery from fatigue), and sleep length [23]. We found that forest bathing significantly improved the sleepiness on rising and the feeling refreshed (recovery from fatigue) assessed by the OSA-MA. On the other hand, urban area walking did not improve the subjective sleep quality. We previously found that forest bathing significantly increased sleep time [3]. Morita et al [5] reported that two hours of forest walking improved nocturnal sleep conditions for individuals with sleep complaints, possibly as a result of exercise and emotional improvement. The forest bathing/shinrin-yoku also improved depressive symptoms in the POMS test confirmed the previous findings [3, 4, 9–16].

To control for the effects of alcohol, the subjects did not consume alcohol during the study period. It has been reported that physical activity affects mental health and depression biomarkers [27]; therefore, we have to control the effect of physical activity. To control the effect of physical activity, subjects walked the same distance (5 km/day) during the same period in both trips. We also confirmed that there was no significant difference in walking steps, physical activity and lactic acid concentrations in serum between the forest bathing and urban area walking. Ohko et al. [28] reported that the levels of lactic acid in serum in healthy young men are 7.1 ± 2.2 mg/dl (mean ± SD, n = 10). The concentrations are slightly higher than our subjects which are 6.63 ± 1.79 mg/dL (mean ± SD, n = 20) in urban area walking, but at comparable levels.

Since patients with MDD show lower serotonin in serum [17–22], sleep disorders and depressive symptoms [24, 25] and forest bathing could increase serotonin in serum and improve depressive symptoms and subjective sleep quality, it suggests that forest bathing may have potential preventive effect on MDD. However, this effect needs to confirm in healthy female subjects and in the patients with MDD in the future studies.

Some limitations of our study must be acknowledged. First, only healthy male subjects were investigated in the present study, healthy female subjects and the patients with MDD also should be investigated. In fact, we first conducted the study in health male subjects, then we have planned to conduct the studies in female subjects and the subjects who are diagnosed with depression in the following years. However, due to COVID-19 pandemic, experiments in female subjects and patients with depression were postponed. We are planning to conduct these studies in the next year. Second, the number of subjects was only 20 and more subjects should be investigated. However, since the forest bathing study measures many indicators in a field study, the number of subjects is limited in one experiment. In addition, all subjects have to stay at the same hotel for control the diet; however, there is no bigger hotel in Akasawa Shizen Kyuyourin and the hotel has a limited capacity, with a maximum of 20 subjects. In addition, it is necessary to keep a distance between the subjects when walking in the city, and when there are many people, the traffic will be affected; therefore, a permission from the city authorities cannot be obtained. In consideration of various situations, the number of subjects was finally limited to 20. Third, we did not measure tryptophan levels in serum. In fact, tryptophan in serum is also an important biomarker in the evaluation of depression [21]. We will measure tryptophan levels in serum in the next study.

Although this forest bathing study was conducted in the Japanese forest environment, forest bathing is possible all over the world in similar forest environments. In fact, as a method of stress management, promoting health and/or preventing diseases, forest bathing/shinrin-yoku that originated in Japan is spreading all over the world now and becoming a focus of public attention in the world [1–3, 6, 7, 29]. This is the generalizability of this study.

Despite the above limitations, this study has the following strengths.

1. This study revealed for the first time that forest bathing increases the levels of serotonin in serum in middle aged male subjects.

2. This study was conducted in the birthplace of forest bathing in Japan, Akasawa Shizen Kyuyourin. This forest is one of the three most beautiful forests in Japan [1, 2]. We have conducted several forest bathing experiments in this forest so far [8, 13–16]. A good forest environment ensured the reliability of the data.

3. By adopting a crossover research design, this study eliminates order bias and improves the accuracy of statistical analysis.

## Conclusions

Our study indicated that forest bathing produced a significant

(1) increase in level of serotonin in serum,
(2) improvement in subjective sleep quality (feeling refreshed, recovery from fatigue) assessed by the OSA-MA.
(3) decreases in negative moods such as fatigue and increase in feelings of vigor in the POMS test in middle-aged males.

Taken together, the forest bathing/shinrin-yoku program induced significant positive effects on serotonin in serum, depressive symptoms and subjective sleep quality in middle-aged males. These findings suggested a potential preventive effect on depression (depressive status) in male subjects.

## Declaration

### Availability of data and material

The datasets used and analyzed during the current study are available on reasonable request to the corresponding author.

### Authors’ information

Qing Li, MD, PhD, clinical professor, Department of Rehabilitation Medicine, Graduate School of Medicine, Nippon Medical School, Tokyo, Japan

Hiroko Ochiai, MD, PhD, Department of Plastic and Reconstructive Surgery, Laboratory of Regenerative Medicine, Division of Hearing and Balance Disorder, National Institute of Sensory Organs, National Hospital Organization Tokyo Medical Center

Toshiya Ochiai, Forest Baubiologie Studio Inc., Tokyo, Japan

Norimasa Takayama, PhD, Forestry and Forest Products Research Institute, Forest Research and Management Organization, Tsukuba, Japan

Shigeyoshi Kumeda, MD, PhD, Nagano Prefectural Kiso Hospital, Nagano prefecture, Japan

Takashi Miura, Agematsu Town Office Industry & Tourism Department, Nagano prefecture, Japan

Yoichiro Aoyagi, MD, PhD, professor, Department of Rehabilitation Medicine, Graduate School of Medicine, Nippon Medical School, Tokyo, Japan

Michiko Imai, MD, PhD, the president of INFOM (International Society of Nature and Forest Medicine), Tokyo, Japan

## References

[1] Li Q. Shinrin-yoku. The Art and Science of Forest Bathing – How Trees Can Help You Find Health and Happiness. Penguin Random House UK, London, UK, 2018; pp. 1–320.

[2] Li Q. Forest Bathing. The Japanese Art and Science of Shinrin-yoku. Viking Books, New York, USA, 2018; pp. 1–320.

[3] Li Q. Forest Medicine. In: Li, Q. (ed): Forest Medicine. Nova Science Publishers, Inc., NY, USA, 2012; pp. 1–316.

[4] Li Q, Morimoto K, Nakadai A, Inagaki H, Katsumata M, Shimizu T, . Forest bathing enhances human natural killer activity and expression of anti-cancer proteins. Int J Immunopathol Pharmacol. 2007;20:3–8. 17903349 10.1177/03946320070200S202

[5] Morita E, Imai M, Okawa M, Miyaura T, Miyazaki S. A before and after comparison of the effects of forest walking on the sleep of a community-based sample of people with sleep complaints. Biopsychosoc Med. 2011;5:13. doi: 10.1186/1751-0759-5-13. 21999605 PMC3216244

[6] Li Q. SHINRIN-YOKU - L’art et la science du bain de forêt - Comment la forêt nous soigne. Editions First, Paris, France, 2018; pp. 1–320 (in French).

[7] Li Q. Shinrin-yoku. El poder del bosque. Shinrin-Yoku-Cómo encontrar la salud y la felicidad a través de los árboles- Roca Editorial, Spain, 2018; pp. 1–312 (in Spanish).

[8] Li Q, Morimoto K, Kobayashi M, Inagaki H, Katsumata M, Hirata Y, . Visiting a forest, but not a city, increases human natural killer activity and expression of anti-cancer proteins. Int J Immunopathol Pharmacol. 2008;21:117–27. 18336737 10.1177/039463200802100113

[9] Li Q, Morimoto K, Kobayashi M, Inagaki H, Katsumata M, Hirata Y, . A forest bathing trip increases human natural killer activity and expression of anti-cancer proteins in female subjects. J Biol Regul Homeost Agents. 2008;22:45–55. 18394317

[10] Li Q, Kobayashi M, Inagaki H, Hirata Y, Hirata K, Li YJ, . A day trip to a forest park increases human natural killer activity and the expression of anti-cancer proteins in male subjects. J Biol Regul Homeost Agents. 2010;24:157–65. 20487629

[11] Li Q. Effect of forest bathing trips on human immune function. Environ Health Prev Med. 2010;15(1):9–17. doi: 10.1007/s12199-008-0068-3. 19568839 PMC2793341

[12] Li Q, Otsuka T, Kobayashi M, Wakayama Y, Inagaki H, Katsumata M, . Acute effects of walking in forest environments on cardiovascular and metabolic parameters. Eur J Appl Physiol. 2011;111:2845–53. 21431424 10.1007/s00421-011-1918-z

[13] Li Q, Kobayashi M, Kumeda S, Ochiai T, Miura T, Kagawa T, . Effects of Forest Bathing on Cardiovascular and Metabolic Parameters in Middle-Aged Males. Evid Based Complement Alternat Med. 2016;2016:2587381. doi: 10.1155/2016/2587381. 27493670 PMC4963577

[14] Song C, Ikei H, Kobayashi M, Miura T, Taue M, Kagawa T, . Effect of Forest Walking on Autonomic Nervous System Activity in Middle-Aged Hypertensive Individuals: A Pilot Study. Int J Environ Res Public Health. 2015;12(3):2687–99. doi: 10.3390/ijerph120302687. 25739004 PMC4377926

[15] Ochiai H, Ikei H, Song C, Kobayashi M, Takamatsu A, Miura T, . Physiological and psychological effects of forest therapy on middle-age males with high-normal blood pressure. Int J Environ Res Public Health. 2015;12(3):2532–42. doi: 10.3390/ijerph120302532. 25809507 PMC4377916

[16] Ochiai H, Ikei H, Song C, Kobayashi M, Miura T, Kagawa T, . Physiological and Psychological Effects of a Forest Therapy Program on Middle-Aged Females. Int J Environ Res Public Health. 2015;12(12):15222–32. doi: 10.3390/ijerph121214984. 26633447 PMC4690920

[17] Tao R, Li H. High serum uric acid level in adolescent depressive patients. J Affect Disord. 2015;174:464–6. doi: 10.1016/j.jad.2014.12.031. 25553407

[18] Baidina TV, Trushnikova TN, Danilova MA. Interferon-induced depression and peripheral blood serotonin in patients with multiple sclerosis. Zh Nevrol Psikhiatr Im S S Korsakova. 2018;118(8. Vyp. 2):77–81. doi: 10.17116/jnevro201811808277. [Article in Russian; Abstract in English]. 30160672

[19] Manoharan A, Rajkumar RP, Shewade DG, Sundaram R, Muthuramalingam A, Paul A. Evaluation of interleukin-6 and serotonin as biomarkers to predict response to fluoxetine. Hum Psychopharmacol. 2016;31(3):178–84. doi: 10.1002/hup.2525. 27018372

[20] Jaworek AK, Jaworek M, Makara-Studzińska M, Szafraniec K, Doniec Z, Szepietowski J, . Depression and Serum Content of Serotonin in Adult Patients with Atopic Dermatitis. Adv Exp Med Biol. 2020 Jan 9. doi: 10.1007/5584_2019_470. 31916233

[21] Almeida-Montes LG, Valles-Sanchez V, Moreno-Aguilar J, Chavez-Balderas RA, García-Marín JA, Cortés Sotres JF, . Relation of serum cholesterol, lipid, serotonin and tryptophan levels to severity of depression and to suicide attempts. Psychiatry Neurosci. 2000;25(4):371–7. PMC1407732 11022402

[22] Moroianu LA, Cecilia C, Ardeleanu V, Pantea Stoian A, Cristescu V, . Clinical Study of Serum Serotonin as a Screening Marker for Anxiety and Depression in Patients with Type 2 Diabetes. Medicina (Kaunas). 2022;58(5):652. doi: 10.3390/medicina58050652. 35630069 PMC9146121

[23] Yamamoto Y, Tanaka H, Takase M, Yamazaki K, Azumi K, Shirakawa S. Standardization of revised version of OSA sleep inventory for middle age and aged. Brain Science and Mental Disorder. 1999;10(4):401–9. [in Japanese].

[24] Elhwuegi AS. Central monoamines and their role in major depression. Prog Neuropsychopharmacol Biol Psychiatry. 2004;28(3):435–51. 15093950 10.1016/j.pnpbp.2003.11.018

[25] Zhu DM, Zhang C, Yang Y, Zhang Y, Zhao W, Zhang B, . The relationship between sleep efficiency and clinical symptoms is mediated by brain function in major depressive disorder. J Affect Disord. 2020;266:327–37. doi: 10.1016/j.jad.2020.01.155. 32056895

[26] Eddie D, Bentley KH, Bernard R, Yeung A, Nyer M, Pedrelli P, . Major depressive disorder and insomnia: Exploring a hypothesis of a common neurological basis using waking and sleep-derived heart rate variability. J Psychiatr Res. 2020;123:89–94. doi: 10.1016/j.jpsychires.2020.01.015. 32044591 PMC7047553

[27] Takahashi M, Lim PJ, Tsubosaka M, Kim HK, Miyashita M, Suzuki K, . Effects of increased daily physical activity on mental health and depression biomarkers in postmenopausal women. J Phys Ther Sci. 2019;31(4):408–13. doi: 10.1589/jpts.31.408. 31037019 PMC6451947

[28] Ohko H, Umemoto Y, Sakurai Y, Araki S, Kojima D, Kamijo Y, . The effects of endurance exercise combined with high-temperature head-out water immersion on serum concentration of brain-derived neurotrophic factor in healthy young men. Int J Hyperthermia. 2021;38(1):1077–85. doi: 10.1080/02656736.2021.1922761. 34278925

[29] Kotte D, Li Q, Shin WS, Michalsen A. International Handbook of Forest Therapy. Cambridge Scholars Publishing, London, UK, 2019; pp. 1–610.

